# Retraction Note: Effect of whey protein-derived decapeptide on mood status and blood variables in healthy adults: a randomized, double-blind, placebo-controlled cross-over trial

**DOI:** 10.1007/s00394-025-03797-5

**Published:** 2025-09-22

**Authors:** Katsuya Suzuki, Yoriko Okamatsu, Ryo Uchida, Ikuko Sasahara, Masamichi Takeshita, Wataru Sato, Yoshiro Kitahara, Hitoshi Murakami

**Affiliations:** 1 Institute of Food Sciences and Technologies, Ajinomoto Co., Inc, Kanagawa, 210-8681 Japan; 2 Research Institute for Bioscience Products and Fine Chemicals, Ajinomoto Co., Inc, Kanagawa, 210-8681 Japan

**Retraction Note to: European Journal of Nutrition (2024) 63:2789–2799** 10.1007/s00394-024-03464-1

The authors have retracted this article. After publication a thorough review of our data and analysis highlighted significant errors that compromise the validity of our findings. Specifically, we found that the test food we used did not contain the expected active component. Given the extent of these issues, we believe that retracting the article is the most responsible course of action to maintain the integrity of the scientific record.

All authors agree with this retraction.

